# Parenteral Nutrition-Induced Liver Function Complications: Incidence, Risk Factors, and Prognosis

**DOI:** 10.3390/jcm14041220

**Published:** 2025-02-13

**Authors:** Jae Woo Park, Sun Ah Maeng, Sang Gyune Kim, Young Seok Kim, Jeong-Ju Yoo

**Affiliations:** Department of Internal Medicine, Gastroenterology and Hepatology, School of Medicine, Soonchunhyang University, Bucheon 14584, Republic of Korea; pjw1257@naver.com (J.W.P.); msa0032@naver.com (S.A.M.); mcnulty@schmc.ac.kr (S.G.K.); liverkys@schmc.ac.kr (Y.S.K.)

**Keywords:** parenteral nutrition, parenteral nutrition-associated liver disease, liver injury, abnormal liver function test

## Abstract

**Background/Objectives**: Parenteral Nutrition-Associated Liver Disease (PNALD) is a significant complication in patients undergoing parenteral nutrition (PN). This study aims to explore the incidence, risk factors, and outcomes associated with PNALD, including abnormal liver function tests, in patients receiving parenteral nutrition, even in short-term PN recipients. **Methods**: A retrospective analysis of 500 patients receiving PN for at least 3 days at a tertiary medical center was conducted. Liver enzyme levels were monitored for 28 days, and PN duration, comorbidities, and metabolic factors were analyzed to identify independent risk factors of abnormal liver function tests and PNALD. **Results**: This study reported a 24.4% incidence of abnormal liver function tests and an 8.2% incidence of PNALD. Risk factors for abnormal liver function tests included liver disease (OR 2.064, 95% CI 1.224–3.479), infection (OR 1.654, 95% CI 1.075–2.546), PN duration (OR 1.035, 95% CI 1.014–1.056), and PN calories (OR 1.001, 95% CI 1.000–1.002). Significant PNALD risk factors comprised liver disease (OR 3.623, 95% CI 1.670–7.858), lung disease (OR 3.648, 95% CI 1.615–8.240), recent surgery (OR 3.719, 95% CI 1.645–8.407), PN duration (OR 1.041, 95% CI 1.016–1.068), total cholesterol (OR 1.005, 95% CI 1.000–1.010), and HDL-cholesterol (OR 1.012, 95% CI 1.001–1.023). The majority of PNALD cases (85.3%) showed improvement with PN modification or cessation. **Conclusions**: This study underscores that abnormal liver function tests and PNALD risks can emerge with short-term PN use. Identifying and addressing patient-specific risk factors is vital for predicting and preventing PNALD onset.

## 1. Introduction

Parenteral nutrition, a crucial intervention for hospitalized patients unable to meet nutritional needs orally or enterally, is often associated with various complications [1,2]. Among these, prevalent issues include hyperglycemia, electrolyte imbalances, infections, and abnormalities in liver function tests. Elevated liver enzymes, particularly concerning, may indicate severe hepatic dysfunction [3,4]. This concern is notable in patients without pre-existing liver disease, as parenteral nutrition can induce damage through mechanisms such as steatosis, cholestasis, and gallbladder sludge formation [5,6,7].

A specific manifestation within this context is Parenteral Nutrition-Associated Liver Disease (PNALD), characterized by hepatic dysfunction resulting from parenteral nutrition use, particularly attributed to failures in intestinal absorption or digestion processes [8,9,10]. PNALD is defined by an elevation of at least 1.5 times the upper limit of normal in any two of the following liver enzymes: alanine aminotransferase (ALT), aspartate aminotransferase (AST), or alkaline phosphatase (ALP). The diagnosis requires the exclusion of other potential causes of liver dysfunction [11]. The incidence of PNALD is known to rise with prolonged parenteral nutrition use, typically becoming evident after 14 days of treatment [12]. However, liver enzyme elevations have also been observed in patients undergoing shorter durations of parenteral nutrition, suggesting a need for a more comprehensive investigation into this phenomenon [13].

Prior research has reported the incidence of PNALD in adults to range from 15 to 40% [14], but recent studies suggest a potentially higher prevalence, ranging from 46 to 59.1% [13,15]. A comprehensive approach, covering a broader spectrum of patient durations on parenteral nutrition, is, thus, essential for a more accurate assessment of PNALD prevalence and identification of risk factors.

Continuous efforts have been made to identify risk factors for the prevention of PNALD. Recent studies have focused on predicting PNALD at an early stage of PN administration and assessing patient-specific factors to improve risk stratification and early intervention strategies [13,15,16,17].

Furthermore, attributing liver dysfunction in patients undergoing parenteral nutrition is intricate, as liver enzyme elevations can result from various factors, including infections, independent of the nutrition method [18,19,20]. Discerning specific risk factors for PNALD relies on distinguishing liver function test abnormalities specifically associated with PNALD from those arising from other causes.

This study aims to rigorously examine and identify the risk factors for PNALD by incorporating a diverse patient population undergoing varying durations of parenteral nutrition and carefully differentiating between liver function abnormalities specific to PNALD.

## 2. Materials and Methods

### 2.1. Study Design

We conducted a retrospective, single-center cohort study at a tertiary medical center, encompassing 500 patients who received parenteral nutrition from December 2022 to February 2023. Eligible participants had a minimum parenteral nutrition duration of three days. The cohort consisted of individuals receiving exclusive parenteral nutrition and those utilizing a combination of enteral and parenteral nutrition. Liver enzyme levels were monitored for 28 days according to the duration of parenteral nutrition, and the etiology of enzyme elevation was assessed to determine whether it was attributable to parenteral nutrition or other factors. Furthermore, risk factors associated with liver enzyme elevation were analyzed and compared among patients. The study research protocol was approved by the Institutional Review Boards (IRBs) of Soonchunhyang University Bucheon Hospital (IRB number: SCHBC 2024-01-020-001, registered on 5 March 2024). The study protocol conformed to the ethical guidelines of the World Medical Association Declaration of Helsinki. Patient consent was waived due to the retrospective design.

### 2.2. Patient Selection and Data Collection

Eligible patients were identified through a comprehensive review of medical records. Patients who received PN for at least three days during the study period were included, while those with baseline liver function test abnormalities were excluded. Collected data included demographic information (age, gender), anthropometrics (body mass index [BMI], height, weight), and detailed medical histories (past medical history, comorbidities, surgical history).

Comorbidities were systematically assessed, including liver disease, hypertension, diabetes, cardiovascular disease, lung disease, malignancy, and kidney disease. For patients with hepatocellular carcinoma (HCC), the condition was categorized under both liver disease and malignancy, while lung cancer was classified under both lung disease and malignancy. Similarly, renal cell carcinoma was included under both kidney disease and malignancy to ensure accurate classification of comorbid conditions. Additionally, a recent history of surgery performed under general anesthesia was recorded as a potential influencing factor.

Clinical data encompassed the department of admission, primary diagnosis, concurrent infections, and medication use. Baseline laboratory assessments, conducted before initiating parenteral nutrition, covered a range of parameters, including complete blood count (CBC), C-reactive protein (CRP), liver function tests (AST [aspartate aminotransferase], ALT [alanine aminotransferase], ALP [alkaline phosphatase]), total bilirubin, direct bilirubin, coagulation profile (PT [prothrombin time], INR [international normalized ratio]), lipid panel, serological markers for viral hepatitis (HBsAg [hepatitis B surface antigen], and anti-HCV [antibody to hepatitis C virus]). Follow-up laboratory measurements were obtained up to 28 days post-initiation of parenteral nutrition. Detailed information regarding the parenteral nutrition regimen (type, duration, caloric content, indications, and administration route—central or peripheral) was meticulously recorded. The occurrence and resolution of PNALD were also documented.

### 2.3. Outcome Definition

Abnormal liver function was characterized by elevated AST or ALT levels exceeding 1.5 times the upper limit of normal or an increase in total bilirubin above the upper limit, accompanied by ALP levels surpassing 1.5 times the upper limit of normal. PNALD was classified into three categories: (1) steatosis type, defined as the presence of steatosis with AST or ALT levels exceeding 1.5 times the upper limit of normal; (2) cholestasis type, characterized by a total bilirubin level above the upper limit of normal and ALP exceeding 1.5 times the upper limit of normal; and (3) mixed type, presenting with features of both steatosis and cholestasis. We conducted a comprehensive assessment to identify potential causes of liver function abnormalities, including PNALD, infection, viral hepatitis, drug-induced liver injury, ischemic hepatitis, alcoholic hepatitis, non-alcoholic steatohepatitis, liver cirrhosis, hepatic malignancy, chronic liver disease, metastatic disease to the liver and biliary tract diseases. PNALD was precisely defined as liver dysfunction attributable to PN, excluding other potential causes such as infections, drug toxicity, or pre-existing liver disease. PNALD was confirmed only in cases where PN was identified as the sole risk factor for liver enzyme elevation. If liver enzyme levels decreased after discontinuation or modification of PN, this provided further support for the diagnosis of PNALD. Patients with pre-existing liver disease were included only if their hospitalization was unrelated to liver disease and they were not experiencing an acute liver condition at the time of this study. To minimize confounding factors, all patients receiving medications known to cause drug-induced liver injury were excluded from the PNALD group. This exclusion criterion ensured that liver dysfunction was specifically attributed to PN rather than the potential hepatotoxic effects of medications.

### 2.4. Statistical Methods

Baseline characteristics were presented using means ± standard deviations for continuous variables and frequencies and percentages for categorical variables. Continuous variables underwent *t*-test comparisons, while chi-square tests were applied to analyze categorical variables. The incidence and progression of abnormal liver function tests and PNALD were assessed through Kaplan–Meier survival analysis. Univariable and multivariable logistic regression analyses were employed to identify risk factors for abnormal liver function tests and PNALD. Variables with a *p*-value of less than 0.05 in the univariable analysis were incorporated into the multivariable model. The statistical analysis was conducted using SPSS software, version 23.0 (IBM Corp., Armonk, NY, USA).

## 3. Results

### 3.1. Baseline Characteristics

Among the 500 patients enrolled in this study, 273 (54.6%) were male, and 237 (45.4%) were female, with a mean age of 68.7 years. The average BMI was 23.2 kg/m^2^, height measured at 160.2 cm, and weight at 60.2 kg. Prevalent comorbidities included hypertension (48.2%), diabetes (30.0%), cardiovascular disease (29.2%), lung disease (27.0%), and malignancy (39.8%). Parenteral nutrition was predominantly utilized in the general surgery department (20.4%), followed by pulmonology (17.6%) and gastroenterology (17.0%). The mean duration of parenteral nutrition use for all patients was 9.1 days. Detailed information on initial laboratory findings and baseline characteristics can be found in Table 1.

### 3.2. Incidence of Abnormal Liver Function Tests

**Figure 1:**
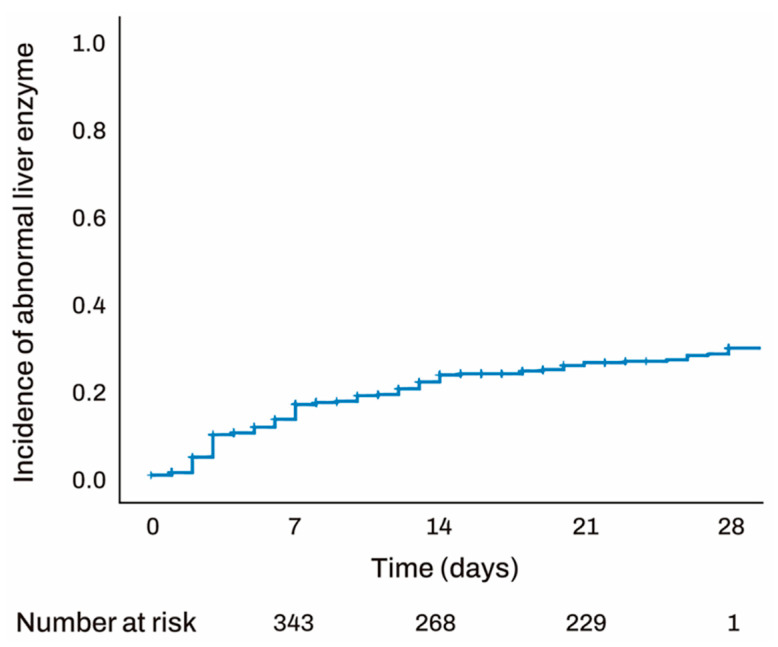
Kaplan–Meier of Abnormal liver function test. Abnormal liver function tests occurred in 45 (40.1%) cases within 3 days, 31 (27.6%) cases between 3 and 7 days, 26 (23.2%) cases between 7 and 14 days, and 20 (17.8%) cases after 14 days.

Among the patients, 112 (24.4%) exhibited abnormal liver function tests during the study period, with an average duration of parenteral nutrition use in this subgroup being 9.1 days. The median time to enzyme elevation was 6.5 days. Abnormal liver function tests occurred in 45 (40.1%) cases within 3 days, 31 (27.6%) cases between 3 and 7 days, 26 (23.2%) cases between 7 and 14 days, and 20 (17.8%) cases after 14 days. The pulmonology department had the highest proportion of patients with abnormal liver function tests (26.7%), representing 34.1% of pulmonology patients, followed by general surgery and hepato-oncology (Table 2). The distribution of abnormal liver function tests in relation to the duration of parenteral nutrition use is visually depicted in Figure 1.

### 3.3. Incidence of PNALD

**Figure 2:**
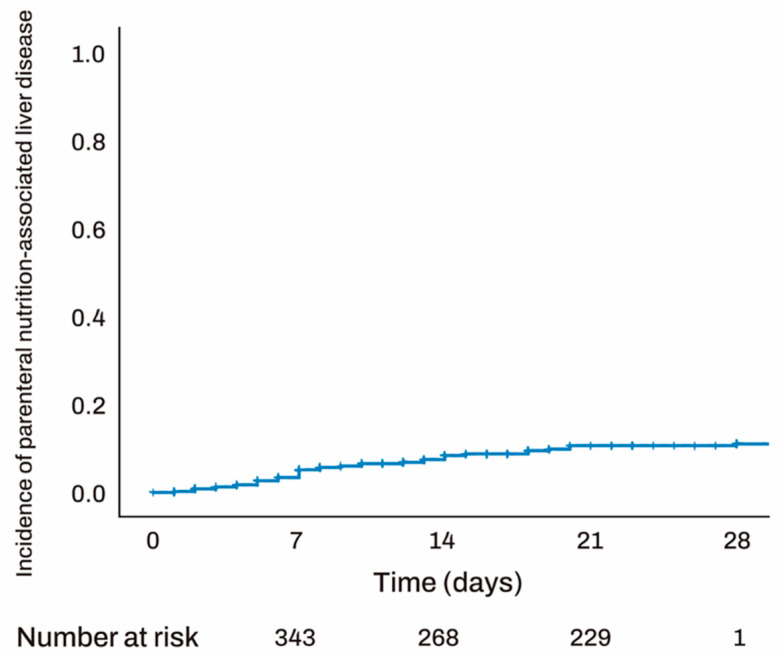
Kaplan–Meier of PNALD accumulation. PNALD occurred in 6 (14.6%) cases within 3 days, 15 (36.5%) cases between 3 and 7 days, 11 (26.8%) cases between 7 and 14 days, and 7 (17.0%) cases after 14 days. PNALD, Parenteral Nutrition-Associated Liver Disease.

In total, 41 (8.2%) patients developed PNALD, with a mean parenteral nutrition duration of 14.5 days and a median time to onset of 7 days. PNALD occurred in 6 (14.6%) cases within 3 days, 15 (36.5%) cases between 3 and 7 days, 11 (26.8%) cases between 7 and 14 days, and 7 (17.0%) cases after 14 days. Among the 41 PNALD cases, 13 (31.7%) were classified as steatosis type, 21 (51.2%) as cholestasis type, and 7 (17.1%) as mixed type. Pulmonology exhibited the highest incidence of PNALD, affecting 13 (31.7%) patients, constituting 14.8% of the pulmonology cohort. General surgery had the second-highest incidence (Table 2). The trend of PNALD in relation to parenteral nutrition duration is illustrated in Figure 2.

In the PNALD group, 28 (68.2%) were male, and 13 (31.8%) were female, with an average age of 69.7 years. The mean BMI was 23.1 kg/m^2^, and the mean height and weight were 158.9 cm and 64.2 kg, respectively. Comorbidities included hypertension (56.1%), diabetes (34.1%), cardiovascular disease (29.3%), lung disease (43.9%), and malignancy (46.3%). Initial laboratory values were within normal ranges, with an average AST of 33 ± 20, ALT of 21 ± 11, and ALP of 99 ± 64. The FIB-4 score was lower in the PNALD group (3.397 ± 4.457) compared to the control group (5.616 ± 12.827). Significant differences between the PNALD group and the control group were observed only in WBC and albumin levels (Table 1).

### 3.4. Risk Factors for Abnormal Liver Function Tests and PNALD

Univariate analysis identified several parameters potentially associated with abnormal liver function tests or PNALD, including sex, age, BMI, comorbidities (liver disease, hypertension, diabetes, cardiovascular disease, lung disease, malignancy, kidney disease), presence of infection, surgery under general anesthesia, and parenteral nutrition factors (supplementation method, PN duration, PN calories), along with initial laboratory findings (HBsAg, anti-HCV, total cholesterol, triglyceride, HDL-cholesterol).

In the multivariate analysis for abnormal liver function tests, liver disease (odds ratio [OR] 2.064, 95% confidence interval [CI] 1.224–3.479, *p* = 0.007), infection (OR 1.654, 95% CI 1.075–2.546, *p* = 0.022), PN duration (OR 1.035, 95% CI 1.014–1.056, *p* = 0.001), and PN calories (OR 1.001, 95% CI 1.000–1.002, *p* = 0.036) emerged as significant risk factors (Table 3). For PNALD, significant risk factors included liver disease (OR 3.623, 95% CI 1.670–7.858, *p* = 0.001), lung disease (OR 3.648, 95% CI 1.615–8.240, *p* = 0.002), surgery under general anesthesia (OR 3.719, 95% CI 1.645–8.407, *p* = 0.002), PN duration (OR 1.041, 95% CI 1.016–1.068, *p* = 0.002), total cholesterol (OR 1.005, 95% CI 1.000–1.010, *p* = 0.060), and HDL (OR 1.012, 95% CI 1.001–1.023, *p* = 0.027). Except for total cholesterol, all other factors were statistically significant (Table 4).

### 3.5. Prognosis of PNALD

Among the 41 patients with PNALD, 35 (85.3%) demonstrated improvement. The average time to resolution was 8.1 days, with a median of 6 days. Improvement was noted in 12 (29.2%) patients after changing the PN type and 18 (43.9%) after PN withdrawal. Furthermore, 10 (24.3%) patients exhibited improvement before 3 days, 11 (26.8%) between 3 and 7 days, 9 (21.9%) between 7 and 14 days, and 5 (12.1%) between 14 and 28 days. For the PNALD patients who did not show improvement, four were lost to follow-up after discharge, and two expired due to complications from underlying diseases despite continued PN for nutritional support. One of the deceased patients was in the intensive care unit in the pulmonology department, and the other was in hemato-oncology.

## 4. Discussion

### 4.1. Summary of Main Findings

Our study revealed that 24.4% of patients exhibited abnormal liver function tests, with 8.2% developing PNALD. In contrast to previous reports suggesting that PNALD onset typically occurs after 14 days of parenteral nutrition, our findings indicated a median onset of abnormal liver function tests and PNALD at 6.5 and 7 days, respectively. Among the 112 patients (24.4%) who exhibited abnormal liver function tests, the mean duration of PN use in this subgroup was 9.1 days, with a median time to enzyme elevation of 6.5 days. Abnormal liver function tests occurred in 45 cases (40.1%) within 3 days, 31 cases (27.6%) between 3 and 7 days, 26 cases (23.2%) between 7 and 14 days, and 20 cases (17.8%) after 14 days. Similarly, PNALD was diagnosed in 41 patients (8.2%), with a mean PN duration of 14.5 days and a median onset of 7 days. The incidence of PNALD occurred in 6 cases (14.6%) within 3 days, 15 cases (36.5%) between 3 and 7 days, 11 cases (26.8%) between 7 and 14 days, and 7 cases (17.0%) after 14 days.

These findings suggest that both PNALD and abnormal liver function tests can occur across the entire duration of PN use rather than being limited to prolonged administration. Furthermore, they highlight that liver dysfunction can develop even with short-term PN use, reinforcing the need for vigilant liver function monitoring regardless of PN duration [12,13,15].

### 4.2. Risk Factor Analysis

Firstly, we identified risk factors for abnormal liver function tests, including underlying liver disease, infection, PN duration, and caloric content. Infections, commonly associated with liver function abnormalities, were categorized separately due to their independent influence. Secondly, our study specified liver disease, lung disease, recent surgery, total cholesterol, HDL, and PN duration as predictors of PNALD.

Excessive energy intake or overfeeding is a significant nutritional risk factor associated with hepatic steatosis or pathological fat accumulation in the liver. This process is mediated by excessive caloric intake-induced hyperinsulinemia, which enhances lipogenesis and suppresses fatty acid oxidation, contributing to hepatic fat accumulation [4,21].

Previous research has highlighted risk factors for PNALD, such as infection, malnutrition, excess fat, carbohydrates, and calories [22,23,24]. Żalikowska-Gardocka M. et al. [13] reported that surgery within 1 month before PN is a risk factor for PNALD, linked to poor health status and a break in enteral feeding. Also, Golucci APBS et al. [25] found that hypertriglyceridemia and hypercholesterolemia are risk factors for PNALD in pediatric patients associated with an excess of accumulated fat.

### 4.3. Risk of Surgery Under General Anesthesia

Surgical interventions, particularly those involving the gastrointestinal tract, can profoundly disrupt the gut–liver axis, predisposing patients to PNALD. Postoperative ileus, bowel ischemia, and alterations in intestinal microbiota are frequent complications that contribute to intestinal stasis and increased gut permeability. This disruption facilitates bacterial translocation and the release of lipopolysaccharides into the portal circulation, which, in turn, activates Kupffer cells, induces hepatic inflammation, and impairs bile flow regulation [26].

Delayed gastrointestinal recovery following surgery often necessitates prolonged PN dependency, further increasing the risk of PNALD. Extended reliance on PN, particularly in the absence of timely enteral nutrition, is associated with hepatic steatosis and cholestasis. Additionally, prolonged fasting suppresses enterohepatic signaling pathways that regulate bile secretion and gut motility, exacerbating bile stasis and compounding the risk of PNALD [21].

### 4.4. Risk of Lung Disease

Lung disease has been identified as a significant risk factor for PNALD in our study, and this finding is supported by emerging evidence linking hypoxia-induced signaling, gut–liver axis dysregulation, and bile acid metabolism to liver disease progression. Chronic or intermittent hypoxia, a hallmark of many pulmonary conditions such as chronic obstructive pulmonary disease (COPD), bronchopulmonary dysplasia (BPD), and acute respiratory distress syndrome (ARDS), has been shown to exert profound effects on hepatic homeostasis through activation of hypoxia-inducible factors (HIFs).

HIF-1α and HIF-2α are central mediators of cellular adaptation to low oxygen availability and play a dual role in liver pathophysiology. HIF-1α activation is associated with acute hypoxic adaptation, while HIF-2α is implicated in chronic hypoxia-driven liver injury, lipid accumulation, and fibrosis. Studies have demonstrated that HIF-2α enhances hepatic lipid synthesis while impairing β-oxidation, thereby promoting hepatic steatosis and fibrosis. Given that hypoxemia is common in patients with chronic lung disease, it is plausible that persistent HIF activation contributes to the pathogenesis of PNALD by exacerbating hepatic metabolic dysfunction and cholestatic injury [27].

Furthermore, the gut–liver axis plays a crucial role in PNALD development, particularly through its impact on bile acid metabolism and intestinal barrier function. The gut epithelium exists under a physiological hypoxic gradient, and alterations in this environment due to systemic hypoxia can disrupt intestinal barrier integrity, increase bacterial translocation, and promote endotoxemia, all of which have been implicated in hepatic inflammation and cholestatic liver disease. Additionally, HIF-2α activation in the intestinal epithelium has been linked to dysregulated bile acid metabolism, leading to altered enterohepatic circulation and impaired bile flow. Notably, disruption of intestinal HIF-2α has been associated with increased bile acid conjugation and metabolic homeostasis, suggesting that chronic HIF activation may contribute to bile acid imbalance in PNALD patients [27].

Given these mechanisms, it is reasonable to hypothesize that patients with pre-existing lung disease experience chronic hypoxemia, leading to sustained HIF activation in both the liver and intestines, thereby exacerbating PNALD progression. The interplay between hypoxia-induced hepatic dysfunction, bile acid dysregulation, and gut–liver axis perturbations provides a compelling explanation for the observed association between lung disease and PNALD [28].

### 4.5. Importance of PN Management

This study underscores the importance of attentive PN management, highlighting the positive correlation between PN duration, caloric content, and PNALD occurrence. However, assessing the role of PN composition proved challenging due to periodic adjustments made by our nutrition support team at Soonchunhyang Bucheon Hospital. This dedicated team comprises doctors, nutritionists, and pharmacists who prudently manage each patient using PN, calculating the necessary calories and components. The periodic changes made by the nutrition support team to the PN used by patients made it challenging to verify the specific effects of PN based on its type and composition.

### 4.6. Strategies to Reduce PNALD Risk

Our study highlights PN duration, caloric intake, comorbidities, and surgical history as key risk factors for PN-associated liver disease (PNALD). Prolonged PN duration and excessive caloric intake have been strongly associated with hepatic steatosis, cholestasis, and metabolic stress, exacerbating PNALD progression [21]. Careful monitoring of energy requirements and gradual caloric escalation can help prevent metabolic overload, while intermittent PN cycling rather than continuous infusion may improve bile flow and reduce hepatic injury [24]. Given that surgical patients often require prolonged bowel rest, minimizing unnecessary PN use and initiating enteral nutrition (EN) as early as feasible are critical strategies to reduce PNALD risk.

Delayed EN has been linked to biliary stasis, gut atrophy, and increased intestinal permeability, all of which contribute to hepatic inflammation [11]. Even minimal enteral feeding (trophic feeding) has been shown to stimulate bile secretion, maintain gut barrier integrity, and prevent bacterial translocation, thereby mitigating PNALD risk [20]. In addition, surgical patients with prolonged PN dependence may experience gut–liver axis disruption and bile acid dysregulation, further exacerbating hepatic injury. Implementing structured EN advancement protocols and considering gut microbiome modulation through probiotic or microbiome-targeted therapies may help reduce systemic inflammation and protect liver function [28].

Patients with pre-existing comorbidities may have an increased susceptibility to PNALD due to baseline hepatic metabolic dysfunction [26]. In particular, chronic hypoxia in lung disease has been linked to HIF-2α-mediated hepatic lipid accumulation and fibrosis, worsening PNALD progression [27]. Optimizing the management of these underlying conditions and monitoring hepatic function closely may help mitigate risk. Additionally, surgical patients requiring prolonged PN are prone to postoperative ileus, bile stasis, and delayed hepatic adaptation, which further increases PNALD susceptibility. Early mobilization, use of prokinetic agents, and structured PN tapering protocols may aid in reducing PN duration and preventing bile stagnation. Frequent liver function monitoring in these high-risk populations is essential for early intervention [21]. Reducing PNALD risk requires a patient-centered, multifaceted approach, emphasizing limited PN duration, early EN transition, and risk factor-specific management.

### 4.7. Future Research Directions

Further research is needed to elucidate the specific mechanisms through which chronic hypoxia and HIF-2α activation contribute to PNALD pathogenesis in patients with lung disease. While previous studies have demonstrated that HIF-2α promotes hepatic steatosis, fibrosis, and metabolic dysfunction, its precise role in PNALD progression and bile acid dysregulation remains incompletely understood. Investigating the differential roles of HIF-1α and HIF-2α in PNALD may provide insight into potential therapeutic targets.

Moreover, the gut–liver axis represents a crucial but underexplored link between lung disease and PNALD. Future studies should examine how systemic hypoxia alters intestinal barrier integrity, bile acid reabsorption, and microbiota composition, ultimately contributing to hepatic dysfunction. Understanding these pathways may help develop interventions that target hypoxia-mediated gut dysbiosis or bile acid metabolism to mitigate PNALD progression.

Finally, therapeutic strategies aimed at modulating HIF signaling warrant further exploration. While HIF inhibition may reduce hepatic lipid accumulation and cholestasis, its systemic effects, particularly on intestinal function and immune response, must be carefully considered. Future studies should investigate whether selective HIF-2α inhibitors or bile acid modulators can be safely and effectively implemented in patients with PNALD and underlying lung disease.

### 4.8. Limitations

This study has several limitations. Firstly, it was conducted over a short period of only three months, which may limit the generalizability of our findings. Due to the importance placed on including all patients receiving PN, extending the study period was challenging as it would have led to an excessively large patient cohort. However, this approach helped mitigate selection bias by encompassing a comprehensive patient population. Secondly, this study was conducted at a single center, which may introduce selection bias. While we accounted for major confounding factors, residual confounding cannot be entirely excluded. Further multicenter, long-term studies are needed to validate our findings and explore additional mechanisms underlying PNALD development.

## 5. Conclusions

In conclusion, our findings indicate a risk of abnormal liver function tests and PNALD even with short-term PN use. Importantly, the occurrence of PNALD can be anticipated and potentially prevented by identifying and addressing patient-specific risk factors.

## Figures and Tables

**Table 1:** Clinical characteristics of the patients.

Characteristic	Total (*n* = 500)	PNALD Group (*n* = 41)	Control Group (*n* = 459)	*p*-Value
Age, years	68.7 ± 13.6	69.7 ± 13.6	68.6 ± 13.7	0.656
Male, *n* (%)	273 (54.6)	28 (68.2)	248 (54.0)	0.070
BMI, kg/m^2^	23.2 ± 8.4	23.1 ± 4.6	23.3 ± 8.7	0.928
Height, cm	160.2 ± 14.4	158.9 ± 19.7	160.4 ± 13.9	0.524
Weight, kg	60.2 ± 16.4	64.2 ± 24.3	59.8 ± 15.5	0.102
Comorbidity				
Hypertension, *n* (%)	241 (48.2)	23 (56.1)	218 (47.5)	0.088
Diabetes, *n* (%)	150 (30.0)	14 (34.1)	136 (29.6)	0.892
Cardiovascular, *n* (%)	146 (29.2)	12 (29.3)	134 (29.2)	0.218
Lung disease, *n* (%)	135 (27.0)	18 (43.9)	117 (25.5)	0.098
Malignancy, *n* (%)	199 (39.8)	19 (46.3)	180 (39.2)	0.906
Laboratory findings				
WBC, 10^3^/μL	10.59 ± 7.68	12.88 ± 10.10	10.39 ± 7.41	0.047
Hb, g/dL	11.0 ± 4.9	11.3 ± 4.9	11.0 ± 5.1	0.690
Platelet, 10^3^/μL	217 ± 116	238 ± 116	215 ± 117	0.227
CRP, mg/dL	6.88 ± 6.99	6.56 ± 6.78	6.91 ± 6.56	0.755
AST, U/L	73 ± 277	33 ± 20	77 ± 289	0.334
ALT, U/L	41 ± 98	21 ± 11	42 ± 102	0.185
ALP, U/L	121 ± 187	99 ± 64	123 ± 194	0.430
FIB-4 score	5.434 ± 12.369	3.397 ± 4.457	5.616 ± 12.827	0.272
Albumin, g/dL	3.3 ± 1.4	3.9 ± 4.5	3.3 ± 0.7	0.003
Total bilirubin, mg/dL	1.40 ± 3.73	0.86 ± 0.37	1.45 ± 3.89	0.332
Direct Bilirubin, mg/dL	0.48 ± 1.73	0.09 ± 0.15	0.51 ± 1.80	0.135
PT, INR	1.15 ± 0.39	1.09 ± 0.23	1.16 ± 0.40	0.333

PNALD, Parenteral Nutrition-Associated Liver Disease; BMI, body mass index; FIB-4, fibrosis-4; PT, prothrombin time; INR, international normalized ratio.

**Table 2:** Department of PNLAD and abnormal liver function test.

	**Department**																	
	**CS**	**EN**	**EY**	**GS**	**MC**	**MG**	**MH**	**MI**	**MN**	**MP**	**NP**	**NR**	**NS**	**OS**	**PR**	**PS**	**UR**	**Total**
PNALD	1/6	0/1	0/2	6/102	2/24	3/85	3/64	1/15	2/59	13/88	0/1	1/7	4/19	1/6	4/12	0/3	0/6	41/500
(%)	16.7	0.0	0.0	5.9	8.3	3.5	4.7	6.7	3.4	14.8	0.0	14.3	21.1	16.7	33.3	0.0	0.0	8.2
	**Department**																	
	**CS**	**EN**	**EY**	**GS**	**MC**	**MG**	**MH**	**MI**	**MN**	**MP**	**NP**	**NR**	**NS**	**OS**	**PR**	**PS**	**UR**	**Total**
LFT	2/6	0/1	0/2	16/102	8/24	14/85	16/64	3/15	12/59	30/88	0/1	2/7	10/19	2/16	7/12	0/3	0/6	122/500
(%)	33.3	0.0	0.0	15.7	33.3	16.5	25.0	20.0	20.3	34.1	0.0	28.6	52.6	33.3	58.3	0.0	0.0	24.4

PNALD, Parenteral Nutrition-Associated Liver Disease; CS, cardiovascular surgery; EN, ear, nose, and throat; EY, ophthalmology; GS, general surgery; MC, cardiology; MG, gastroenterology; MH, hemato-oncology; MI, infectious disease; MN, nephrology; MP, pulmonology; NP, psychiatry; NR, neurology; NS, neurosurgery; OS, orthopedic surgery; PR, rehabilitation; PS, plastic surgery; UR, urology.

**Table 3:** Risk factor of Abnormal liver function test.

	Univariate		Multivariate	
Variables	OR (95% CI)	*p*-Value	OR (95% CI)	*p*-Value
Sex				
Male	1 (ref)			
Female	0.680 (0.448–1.033)	0.071		
Age	1.000 (0.985–1.015)	0.956		
BMI	0.984 (0.952–1.018)	0.368		
Height	1.004 (0.989–1.020)	0.572		
Weight	1.004 (0.992–1.016)	0.536		
Comorbidity				
Liver disease	2.235 (1.349–3.702)	0.002	2.064 (1.224–3.479)	0.007
Hypertension	1.428 (0.948–2.152)	0.088		
Diabetes	0.969 (0.620–1.515)	0.892		
Cardiovascular disease	1.316 (0.849–2.039)	0.219		
Lung disease	1.453 (0.932–2.265)	0.099		
Malignancy	0.975 (0.642–1.480)	0.906		
Kidney disease	0.688 (0.388–1.221)	0.201		
Infection	1.688 (1.116–2.553)	0.013	1.654 (1.075–2.546)	0.022
Surgery under general anesthesia	0.784 (0.473–1.299)	0.344		
Parenteral nutrition factor				
Central	1 (ref)			
Peripheral	0.753 (0.494–1.148)	0.187		
PN duration	1.038 (1.018–1.059)	<0.001	1.035 (1.014–1.056)	0.001
PN calories	1.001 (1.000–1.001)	0.039	1.001 (1.000–1.002)	0.036
Laboratory findings				
WBC	1.003 (0.978–1.029)	0.814		
Hb	1.054 (0.984–1.130)	0.135		
Platelet	1.000 (0.998–1.002)	0.814		
CRP	1.001 (0.972–1.030)	0.972		
AST	0.999 (0.996–1.001)	0.276		
ALT	0.996 (0.992–1.001)	0.108		
ALP	1.000 (0.999–1.001)	0.474		
FIB4-score	0.994 (0.975–1.014)	0.581		
Albumin	1.059 (0.934–1.201)	0.372		
Total bilirubin	1.018 (0.970–1.069)	0.467		
Direct bilirubin	1.080 (0.972–1.201)	0.152		
PT (INR)	1.223 (0.757–1.976)	0.411		
Total cholesterol	1.000 (0.997–1.003)	0.979		
Triglyceride	1.000 (1.000–1.000)	0.386		
HDL	0.996 (0.987–1.005)	0.386		
HBsAg	1.345 (0.505–3.579)	0.553		
Anti-HCV	0.617 (0.071–5.329)	0.66		

OR, odds ratio; CI, confidence interval; PN, parenteral nutrition.

**Table 4:** Risk factor of PNALD.

	**Univariate**		**Multivariate**	
**Variables**	**OR (95% CI)**	***p*-Value**	**OR (95% CI)**	***p*-Value**
Sex				
Male	1 (ref)			
Female	0.546 (0.276–1.080)	0.082		
Age	1.005 (0.982–1.030)	0.655		
BMI	0.998 (0.959–1.039)	0.927		
Height	0.994 (0.976–1.013)	0.535		
Weight	1.014 (0.997–1.031)	0.104		
Comorbidity				
Liver disease	2.670 (1.317–5.411)	0.006	3.623 (1.670–7.858)	0.001
Hypertension	1.413 (0.742–2.688)	0.293		
Diabetes	1.231 (0.626–2.421)	0.546		
Cardiovascular disease	1.004 (0.497–2.025)	0.992		
Lung disease	2.288 (1.192–4.389)	0.013	3.648 (1.615–8.240)	0.002
Malignancy	1.339 (0.705–2.543)	0.373		
Kidney disease	0.620 (0.236–1.627)	0.332		
Infection	1.683 (0.875–3.235)	0.119		
Surgery under general anesthesia	2.098 (1.070–4.113)	0.031	3.719 (1.645–8.407)	0.002
Parenteral nutrition factor				
Central	1 (ref)			
Peripheral	1.243 (0.645–2.397)	0.516		
PN duration	1.040 (1.016–1.065)	0.001	1.041 (1.016–1.068)	0.002
PN calories	1.000 (0.999–1.002)	0.437		
Laboratory findings				
WBC	1.028 (0.998–1.060)	0.071		
Hb	1.010 (0.961–1.062)	0.695		
Platelet	1.002 (0.999–1.004)	0.227		
CRP	0.993 (0.948–1.040)	0.755		
AST	0.991 (0.979–1.003)	0.129		
ALT	0.989 (0.973–1.004)	0.144		
ALP	0.999 (0.995–1.002)	0.44		
FIB4-score	0.949 (0.875–1.030)	0.209		
Albumin	1.173 (0.961–1.432)	0.117		
Total bilirubin	0.768 (0.475–1.242)	0.281		
Direct bilirubin	0.142 (0.019–1.038)	0.054		
PT (INR)	0.564 (0.185–1.717)	0.313		
Total cholesterol	1.005 (1.001–1.009)	0.021	1.005 (1.000–1.010)	0.06
Triglyceride	1.000 (0.999–1.001)	0.829		
HDL	1.011 (1.002–1.021)	0.015	1.012 (1.001–1.023)	0.027
HBsAg	0.796 (0.178–3.557)	0.765		
Anti-HCV	146213061.0 (0.000)	0.999		

PNALD, Parenteral Nutrition-Associated Liver Disease; OR, odds ratio; CI, confidence interval; PN, parenteral nutrition.

## Data Availability

The datasets used and/or analyzed during the current study are available from the corresponding author upon reasonable request.
